# A quantitative method to assess bacterial adhesion using recombinant bioluminescent *Pseudomonas aeruginosa*

**DOI:** 10.52601/bpr.2021.200043

**Published:** 2021-02-28

**Authors:** Lu Wang, Xinhua Qiao, Lei Gao, Chang Chen, Yi Wan

**Affiliations:** 1 Microbiology Institute of Shaanxi, Xi'an 710043, China; 2 National Laboratory of Biomacromolecules, CAS Center for Excellence in Biomacromolecules, Institute of Biophysics, Chinese Academy of Sciences, Beijing 100101, China; 3 University of Chinese Academy of Sciences, Beijing 100049, China; 4 Beijing Institute for Brain Disorders, Beijing 100069, China

**Keywords:** Bacterial adhesion, PAO1-*lux*, Bioluminescence assay, Medical materials

## Abstract

Bioluminescence technology has been widely used in the field of medical detection. The bioluminescent *lux* reporter system provides a non-invasive platform to monitor bacterial growth and expression in real time. This study aimed to establish a method for detecting bacterial adhesion on the surface of materials, including medical devices, by using recombinant bioluminescent *Pseudomonas aeruginosa* containing a *lux* reporter. By monitoring the growth and bioluminescent properties of the recombinant PAO1-*lux* strain, the optimal test conditions for bacterial adhesion detection *in vitro* were determined to be as follows: an initial inoculation density of 10^5^ to 10^6^ CFU/mL, M9 medium at a pH 6.2, an adhesion time of 6 h, and the collection of adherent bacteria by ultrasonic cleaning. The traditional CFU counting method and the bioluminescence method were compared, and the applicability of the new method was verified by testing the adhesion of bacteria on the surface of various materials. The validated bioluminescent strains could serve as strong candidates to be used as bacterial detection tools in applications such as bacterial adhesion evaluation as well as supplements and alternatives to traditional microbiological testing procedures. In addition, this method has the potential to enable the study of bacterial adhesion on the surface of inanimate objects and living tissues. With the development of this method and its wide applicability, it is expected to become a standard method for the detection of bacterial adhesion and the screening of anti-adhesion materials.

## INTRODUCTION

Medical device-related infections are an important public health concern and account for a large proportion of hospital-acquired infections (Arciola *et al*. 2018; Desrousseaux *et al*. 2013). Bacterial contamination of medical devices is responsible for about 60% to 70% of hospital-acquired infections, particularly in critically ill patients (Bryers 2008; Weinstein and Darouiche 2001). Complications caused by medical device-related infections can lead to increases in postoperative morbidity, mortality and reoperation rate. These complications prolong the treatment cycle and endanger the lives of patients, which result in massive medical and economic burdens (Anderson *et al*. 2014; Badia *et al*. 2017). For example, catheter-associated urinary tract infections have been estimated to account for about 40% of hospital-acquired infections globally, with about 900,000 cases each year in the United States alone, at an annual cost ranging from $296 million to $2.3 billion (Johnson *et al*. 2006; Lo *et al*. 2014). In a study conducted in four European countries, catheter-related bloodstream infections caused an average of more than 1,000 deaths per year in each of these countries, and entailed associated costs of €35 to €164 million totally (Tacconelli *et al*. 2009).

Bacterial adhesion, which paves the way for colonization on solid surfaces, is the initial step in infections caused by medical devices or biomaterials. When bacteria adhere to a surface and grow to a certain density, a variety of virulence factors, including biofilms, can be formed through the quorum sensing system to allow bacteria to survive under adverse environmental conditions and in the presence of antibiotics, and result in drug resistance (Arciola *et al*. 2012, 2018; Swartjes and Veeregowda 2015).

The first prevention and control strategy that was explored to eliminate the contamination of medical devices by targeting bacterial adhesion was the use of antibiotics or biocides and the development of coatings that release antimicrobials or kill micro-organisms by contact (Casey *et al*. 2012; Muñoz-Bonilla and Fernández-García 2012; Onaizi and Leong 2011; Zhao *et al*. 2009). However, numerous clinical trials using these strategies have been performed and yielded conflicting results (Donlan and Costerton 2002). In addition, some researches fear that extensive, long-term use of antibiotics or bactericides on coatings and their release at subinhibitory concentrations could set the stage for the emergence of multidrug-resistant bacteria by virtue of the introduced selection pressure (Stickler 2002). In fact, hospital-acquired infections with multidrug-resistant bacteria account for 65% of all infections with multidrug-resistant organisms (Neubeiser *et al*. 2020), especially in intensive care units (Beilenhoff *et al*. 2017; Darge *et al*. 2019; Russotto *et al*. 2015).

Another effective strategy to prevent the contamination of medical devices by pathogens is to inhibit their adhesion to the surface, which blocks the initial step, rather than killing them directly. The development and utilization of anti-adhesive coatings or materials against bacteria can directly reduce or delay the risk of pathogenic factors, such as biofilms, as a result of decreased bacterial adhesion, which reduces the risk of infections from implanted devices (Hook *et al*. 2012). A variety of medical materials, including nanomaterials and polymers, have been shown to have good anti-bacterial adhesion properties (Campoccia *et al*. 2015; Hsu *et al*. 2013; Serrano *et al*. 2015; Smith *et al*. 2017; Zander and Becker 2017). Furthermore, an increasing number of novel biomaterials and medical devices with antibacterial and anti-bacterial adhesion properties have been developed or are being developed. However, these novel biomaterials, are faced with the testing requirement of material screening, quality control, functional evaluation and product improvement. One particularly important hurdle that needs to be overcome is to determine how to correctly and effectively evaluate the adhesion of bacteria, especially multidrug resistant bacteria, to the surfaces of medical devices.

Even though simplified *in vitro* systems and industrial standard tests have been developed over the last couple of decades to evaluate the anti-adhesion efficacy of medical and non-medical products (Azeredo *et al*. 2017; Catto and Cappitelli 2019), there is still a lack of standardized assays and validated methods to properly assess the activity of anti-adhesion materials (Haney *et al*. 2018). Plate counting, also called colony-forming unit (CFU) counting, is the gold standard of quantitative bacterial detection and is the most traditional, commonly used method. However, the shortcomings of CFU counting are obvious: bacteria need to be cultured for several days, which is time-consuming and labor-consuming; colony aggregation leads to detection errors; bacterial adhesion sites cannot be detected directly; and materials of irregular shape or with different textures cannot be evaluated as a whole. To a large extent, CFU counting depends on manual operation and is impossible to carry out as a high-throughput screening method. Therefore, a series of alternative methods has been developed over the years. Among these methods, adenosine triphosphate (ATP) bioluminescence has been widely used (Kodjikian *et al*. 2008; Mosuela *et al*. 2018). ATP bioluminescence assays depend on the oxidation reaction between ATP and fluorescein in living cells to emit fluorescence. With this method, the number of adherent bacteria is determined indirectly through the proportion relationship between the luminous intensity and the number of microorganisms. Although this method is time-saving, sensitive and easy to operate, the accuracy of this method needs to be improved because it requires an invasive detection of dye that needs to enter cells and non-microbial ATP can interfere with the testing (Shah and Naseby 2015). In recent years, a non-destructive bioluminescence technology has gradually sprung up in the field of medical detection that relies on bacteria engineered to contain the luciferase gene and can determine the number of living bacteria in real time by monitoring the bioluminescence signal of the bacteria. *Pseudomonas aeruginosa* containing the luciferase reporter gene *lux* has been widely used in the fields of bacterial colonization tracking, tumor localization, environmental pollutant toxicity detection, food safety monitoring, and quality control (Avci *et al*. 2017; Thorn *et al*. 2007; Yousuf *et al*. 2016). Through methodological evaluations, bioluminescent methods have shown to be equivalent, if not better, than the traditional plate counting method and the ATP determination method. In addition, bioluminescent methods may be more precise than the ATP determination method, the bioluminescent *lux* reporter system provides a non-invasive platform to monitor bacterial growth and expression in a real-time fashion (Shah and Naseby 2015).

*Lux*-labeled *P. aeruginosa* has been used to evaluate the antibacterial activity of wound dressing *in vitro* and to study the mouse model of catheter-related infections (Kadurugamuwa *et al*. 2003; Thorn *et al*. 2007; Yu *et al*. 2017). However, there is limited research on the establishment of a standard detection method *in vitro* using the bioluminescent *P. aeruginosa* strain to study bacterial adhesion. Most of the studies use bioluminescence to supplement traditional methods, but there has been no systematic research comparing these methodologies. In addition, bioluminescence methods have few applications to the evaluation of bacterial adhesion on medical devices. Furthermore, the current evaluation methods of bacterial adhesion *in vitro* are unable to meet the needs of various materials and lack standardization. Therefore, the purpose of this study is to establish a set of *in vitro* standardized evaluation procedures that can be used to detect the initial adhesion of bacteria on the surfaces of different medical materials simultaneously. Using a constructed recombinant bioluminescent *P. aeruginosa* strain with the *lux* gene marker and through the optimization of external environmental factors and detection window period, different materials of medical devices were selected for testing and comparison. The effectiveness of this newly established method was verified by using surfactant (Triton-X) that has been proven to have inhibitory effects on bacterial adhesion (Ishikawa *et al*. 2012; Liu *et al*. 2019; Neu 1996). In addition, the results of bioluminescence and traditional CFU counting were compared and analyzed to evaluate the equivalence of the two methods. As a supplement or replacement to conventional / traditional microbiological test procedures, the newly established, optimized and validated bioluminescence detection method provides a good experimental basis for its use to detect bacterial adhesion on the surface of medical devices. Furthermore, this study provides data to support the application of bioluminescence technology in rapid, quantitative analysis of microorganisms and real-time monitoring.

## RESULTS

### Characteristics of the recombinant bioluminescent PAO1-*lux* strain

**Figure 1:**
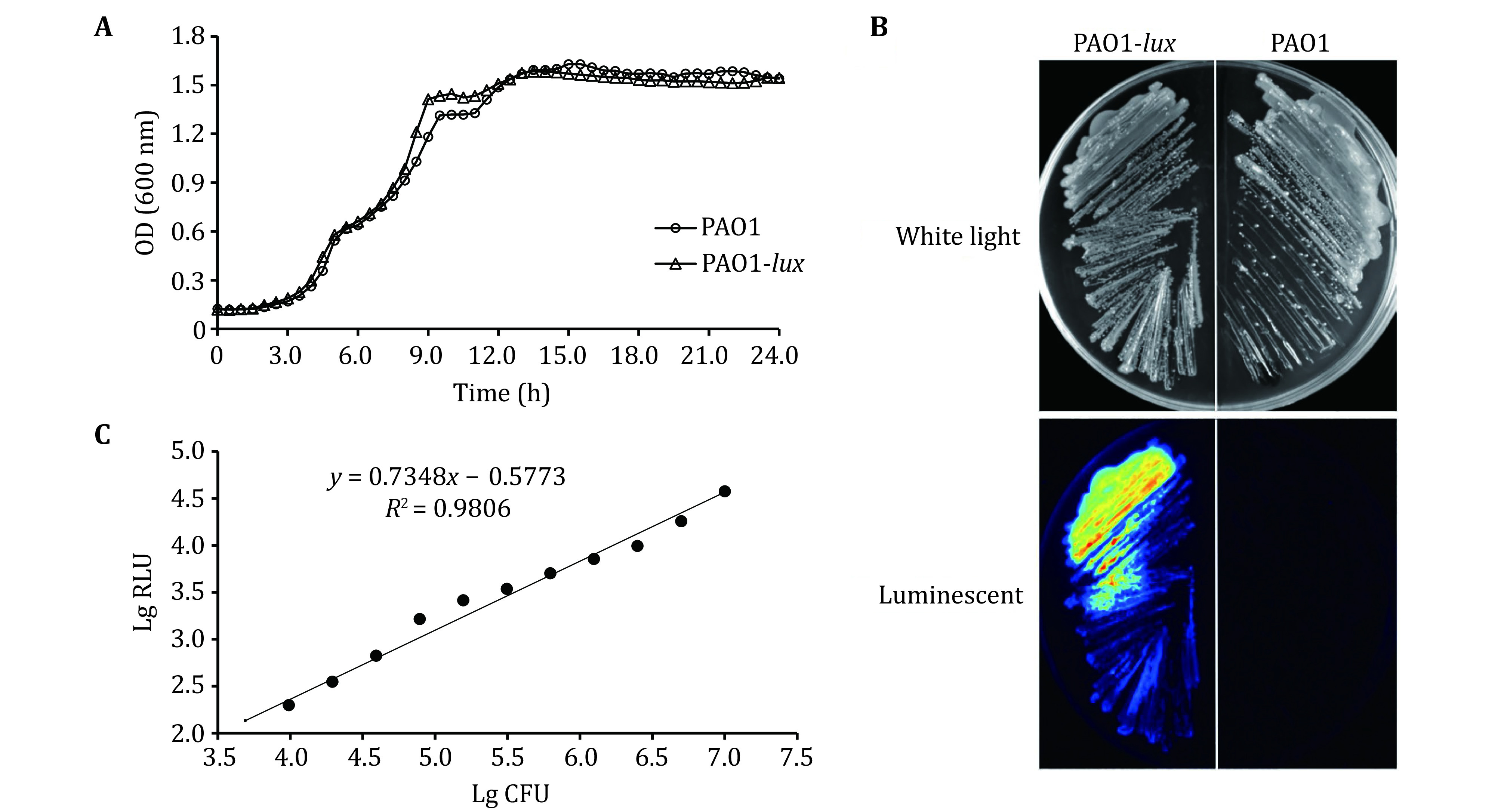
Characteristics of the bioluminescent PAO1-*lux* strain. **A** Growth curves of the PAO1-*lux* and PAO1 strains. Bacteria were cultured in LB medium on 96-well plate for 24 h at 37 °C. The OD at 600 nm was measured at every 0.5 h time points. The data were representative of three independent experiments with similar results. **B** Light detection of the PAO1-*lux* and PAO1 strains using a Bio-Rad imaging system under white light and luminescent modes. Bacteria were cultured on LB plates for 24 h. **C** Light detection of the PAO1-*lux* strain cultured *in vitro* using a multi-mode microplate reader. Data are reported as relative light units (RLU) and wells with sterile saline solution were used to determine the background value, which was subtracted from the bioluminescence values from the wells with bacteria. The corresponding bacterial counts were determined by CFU counting. The data are mean values of three independent experiments

In order to investigate whether the growth of the recombinant bioluminescent strain was affected by the introduction of the reporter gene, the standard PAO1 strain and the recombinant PAO1-*lux* strain were cultured under the same conditions. The OD at 600 nm was measured at the indicated time points over 24 h and the growth curves are shown in Fig. 1A. There was no significant difference in the growth rate of the two strains. The growth of the PAO1-*lux* strain in the logarithmic growth phase and stationary phase basically coincided with that of the standard strain. The colony morphology and bioluminescent property of the PAO1-*lux* strain were photographed using a Bio-Rad imaging system. In the white light mode, both the standard PAO1 strain and the recombinant PAO1-*lux* strain were visible. In the luminescent mode, the PAO1 strain was invisible, but the PAO1-*lux* luminous colony was clearly visible (Fig. 1B). In addition, the bacterial surface structures of the PAO1-*lux* and the PAO1 strains were observed by scanning electron microscopy (SEM), which found that there were no significant differences in the surface structures, such as bacterial flagella, between the two strains suggesting that the introduction of the *lux* gene had no adverse effect on bacterial adhesion (supplementary Fig. S1).

Relative light unit (RLU) measurements were determined for each serial dilution of the PAO1-*lux* strain that was cultured in LB medium to logarithmic phase. The relative production of bioluminescence was compared to CFU counts for the PAO1-*lux* strain to determine the linear range of the bioluminescent assay. When the CFU count was in the range of 10^4^ to 10^7^, there was a good linear relationship between RLU and CFU, *R*^2^ = 0.9806, as shown in Fig. 1C. When the CFU count was below 10^3^, the bioluminescence signal remained constant at baseline and was undistinguishable from the control group without bacteria, which indicated that the lower limit of detection for RLU measurements was about 10^3^ cell/mL. The bioluminescence readings were found to decrease at a linear rate with increasing dilution factor, which means that as the number of cells decreased, the production of bioluminescence decreased at an equal rate (Shah and Naseby 2015).

### Bioluminescence stability of the PAO1-*lux* strain

The PAO1-*lux* and PAO1 strains were cultured to logarithmic phase and serial dilutions were inoculated into a 96-well plate, which was photographed using a Bio-Rad imaging system in white light and luminescent modes. The plate was observed at the 1st, 3rd, and 7th day. The OD at 600 nm and luminous intensity (RLU) were detected by a Multi-Mode Reader (Synergy HTX, Biotek) at the same time. Compared with the standard strain, the PAO1-*lux* strain maintained the bioluminescent property under a certain concentration and time range (supplementary Fig. S2). The PAO1-*lux* strain was cultured for ten consecutive generations and each generation was preserved. In addition, the ten generations of the strain were cultured to stationary phase and serial dilutions of each generation were inoculated into a 96-well plate for imaging and detection. The results showed that the bioluminescent property of the PAO1-*lux* strain was still consistent after ten passages with that of the initial generation and that there was a good linear relationship between the OD value at 600 nm and the RLU (*R*^2^ = 0.9852; supplementary Fig. S3). Because the *lux* gene was integrated into the bacterial chromosome in the form of a single copy, the PAO1-*lux* strain had more stable bioluminescence, which results in it having more advantages in bioluminescent detection and standard method development.

### Effect of culture conditions on growth and bioluminescence of the PAO1-*lux* strain

**Figure 2:**
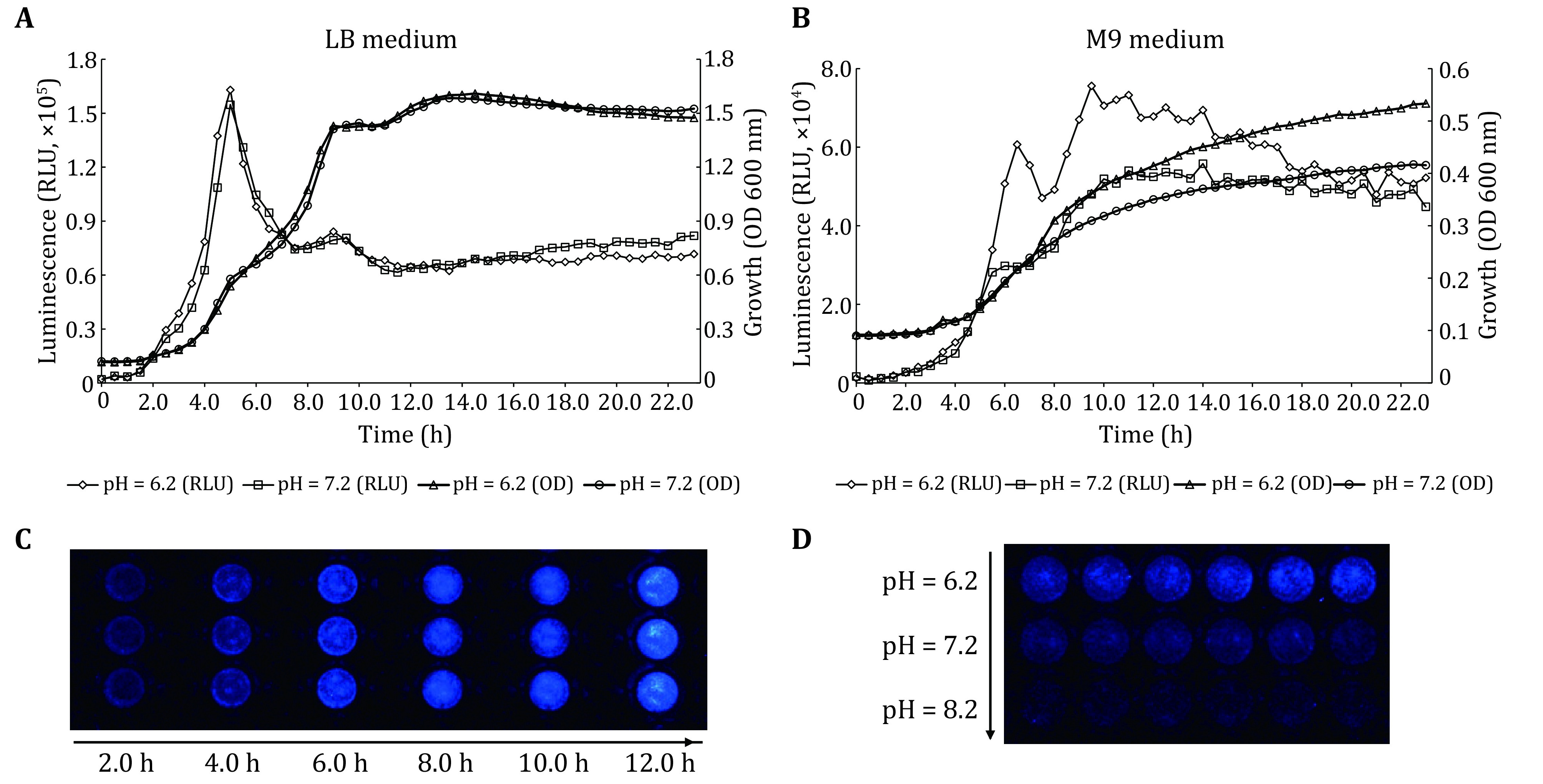
Characteristics of the bioluminescence and growth curves of the PAO1-*lux* strain under different nutritional conditions and different pH conditions. **A** The PAO1-*lux* strain was cultured at 37 °C for 24 h in LB medium with the pH conditions of 6.2 and 7.2. The OD and RLU values of the 96-well microplate were detected by a Multi-Mode Reader every 0.5 h. **B** The bioluminescence pictures of the PAO1-*lux* strain at a single time point for 6 h were collected using a Bio-Rad imaging system. **C** The PAO1-*lux* strain was cultured at 37 °C for 24 h in M9 medium with the pH conditions of 6.2 and 7.2. The OD and RLU values of the 96-well microplate were detected by a Multi-Mode Reader every 0.5 h. **D** The PAO1-*lux* strain was cultured in M9 medium with different pH conditions (pH = 6.2, 7.2 and 8.2) and the picture was observed by the imaging system at 6 h

Environmental factors and conditions, including nutritional conditions, pH value, temperature, culture time, have significant influences on the initial adhesion of bacteria. In order to determine the optimal test conditions of the PAO1-*lux* strain for bacterial adhesion on the surface of materials, the PAO1-*lux* strain was inoculated into a 96-well polystyrene microplate and various conditions were screened. Overnight cultures of the PAO1-*lux* strain were diluted 1:100 in LB or M9 medium and incubated until the OD at 600 nm reached 0.5. The PAO1-*lux* strain was continuously cultured at 37 °C for 24 h under different nutritional conditions (LB or M9 medium) and different pH conditions (pH 6.2 and 7.2). The OD and RLU values of the 96-well microplate were detected by a Multi-Mode Reader every 0.5 h and the bioluminescence pictures at a single time point were collected using a Bio-Rad imaging system. The results in Fig. 2A show that the coincidence degree of the growth curve (solid line) and the bioluminescence curve (dotted line) of PAO1-*lux* was good under the two culture conditions of pH 6.2 and 7.2, indicating that there was no significant difference in growth and bioluminescent properties of the PAO1-*lux* strain in weakly acidic and neutral pH environment for LB medium with adequate nutrition. When cultured for 6 h, the bioluminescent signal of the PAO1-*lux* strain reached the peak and then decreased rapidly, at which time the bacterial growth was in the middle of the logarithmic phase, which indicates that the bioluminescent intensity was not consistent with the logarithmic growth phase. This phenomenon might be related to the promoter used in the construction process of the PAO1-*lux* strain as the intensity of the promoter determines the bioluminescent properties and linear change (Shah and Naseby 2014). The PAO1-*lux* strain used in this study is a strain that integrates the *luxCDABE* fluorescent reporter containing the *algU* gene (which is related to bacterial adhesion and biofilm formation) promoter. The peak of the bioluminescent signal might be related to the strength of the *algU* gene promoter under the nutritional condition of LB medium. The bioluminescent images at different time points during 6 h showed that when the PAO1-*lux* strain was in the logarithmic phase, the bioluminescent intensity increased with the growth of bacteria in the 96-well plate (Fig. 2B).

In M9 medium, the growth curve of the PAO1-*lux* strain was smoother than that of the PAO1-*lux* strain growth curve in LB medium and there was a better correlation between bioluminescent signal and growth. However, there were significant differences between the two pH culture conditions on the growth and bioluminescence of the PAO1-*lux* strain. The results in Fig. 2C show that the trend of the two curves in M9 medium with a pH 6.2 was stronger than that of pH 7.2. Therefore, the weakly acidic and lower nutritional conditions might make it easier for the PAO1-*lux* strain to adhere on a 96-well plate. The bioluminescent signal of the strain reached a high level when it adhered for 6 h to 12 h and then the bioluminescent intensity decreased slightly and gradually tended to be stable with the arrival of the stationary phase of the bacteria. The PAO1-*lux* strain was tested in M9 medium with different pH conditions (pH = 6.2, 7.2 and 8.2) and the photo was observed by the imaging system at 6 h. It was found that the bioluminescent intensity reached the maximum at pH 6.2 (Fig. 2D). Through the above experiments, it was preliminarily determined that the M9 medium with a pH of 6.2 was the most suitable for the culture of bacteria for adhesion detection and the window period of detection is 6 h.

### Evaluation of bacterial adhesion on 96-well microplates by bioluminescence and crystal violet staining method

In order to verify the applicability of the PAO1-*lux* strain in detecting the initial bacterial adhesion on polystyrene microplates, the bacteria were washed and collected under different nutrition conditions (LB or M9 medium) and different pH conditions (pH = 6.2 or 7.2) at 37 °C for 6 h, and then added to the 96-well microplate after resuspension to determine the RLU value by a multi-mode plate reader. At the same time, traditional crystal violet staining method was used to assess. The results determined by the bioluminescence method showed that there was no significant difference (*p* = 0.7024) in the number of bacteria that initially adhered to the LB medium with a pH of 6.2 or 7.2. However, the number of bacteria that adhered to the M9 medium at a pH of 6.2 increased significantly (*p* = 0.0134) compared with the number of bacteria that adhered to the medium at a pH of 7.2. The results of crystal violet staining also showed that the two pH conditions of the LB medium had no significant effect on the initial adhesion of bacteria (*p* = 0.3503) and that the number of bacteria adhering to the 96-well plate was greater under the acidic condition in the M9 medium (supplementary Fig. S4). The results of the two methods for detecting bacterial adhesion in the early stage of infection in 96-well plates were consistent. Compared with CV method, the operation process of bioluminescence detection was relatively simple, less time-consuming and dye free. In addition, the CV method was limited and only suitable for the determination of bacteria adhering to the microplate, but it was not for the measurement of bacterial adhesion on the surface of medical devices with irregular shape. However, the bioluminescence method was not limited by the material or shape of the sample, which enables its use in a number of fields.

### Evaluation of bacterial adhesion on different materials by bioluminescence and CFU counting method

**Figure 3:**
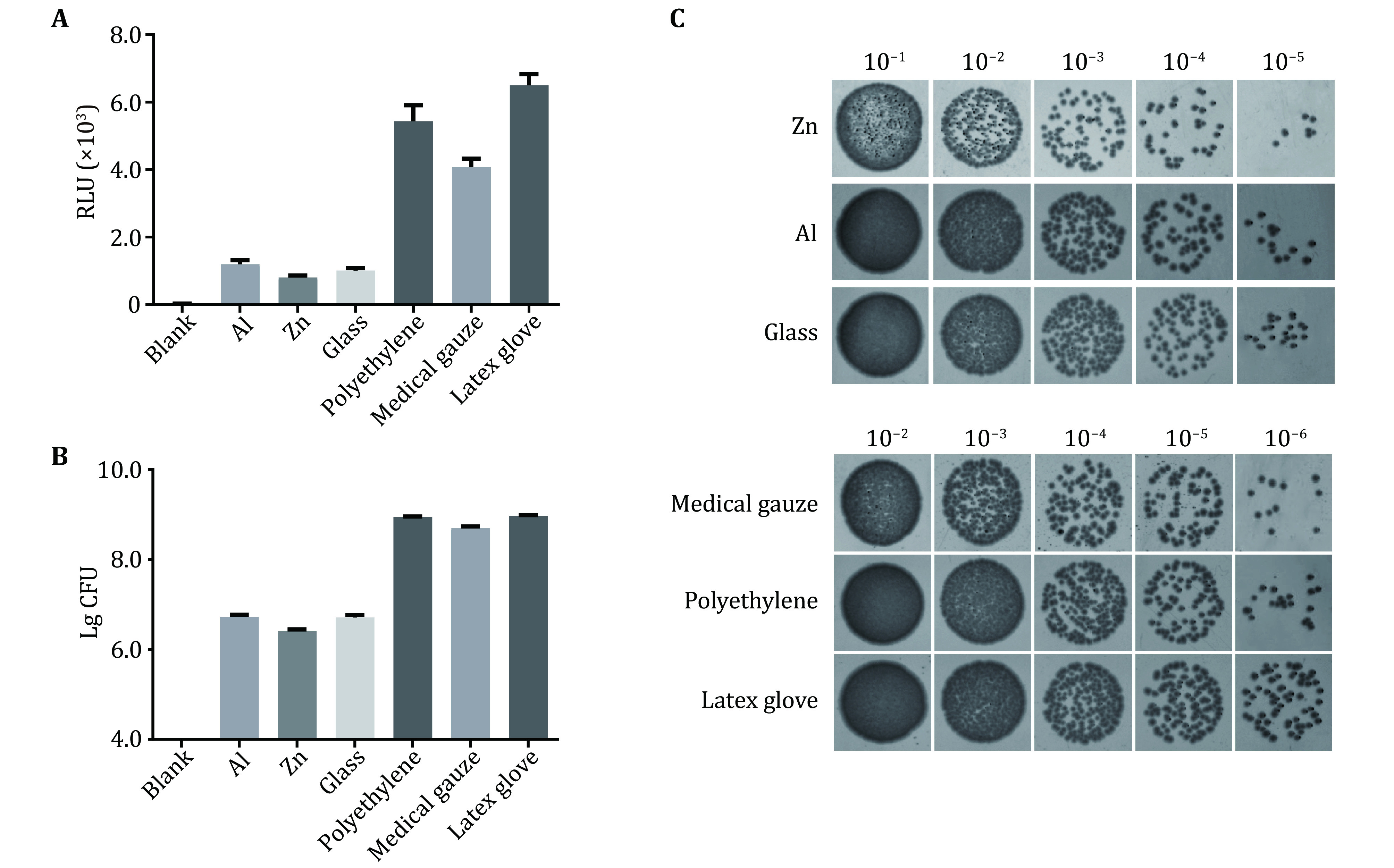
Bacterial adhesion to the six kinds of materials as determined by the bioluminescence method (**A**) and the CFU counting method (**B**, **C**). **A** The RLU values of the PAO1-*lux* strain that adhered to the six kinds of materials. **B** The CFU counts of the PAO1-*lux* strain that adhered to the six kinds of materials. **C** Bacterial adhesion of the PAO1-*lux* strain to the six kinds of materials were determined using the agar dilution assay. Five 10-fold serial dilutions of each sample grown overnight were spotted (10 μL per spot) onto LB agar. The dilution ratio of Zn, Al, glass groups from 10^−1^ to 10^−5^, and medical gauzes, PE gloves, latex gloves groups from 10^−2^ to 10^-6^. The plates were incubated for 16 h to 18 h at 37 °C

In order to further verify the applicability of the bioluminescence method and its equivalence with the traditional method, several samples of different materials were selected for testing. To avoid the errors caused by the surface areas, six kinds of materials, including metal aluminum, metal zinc, glass, disposable PE gloves, medical gauze and medical latex gloves, were cut into thin slices to a size of 2 cm × 2 cm before the test. The surfaces of the materials were covered with bioluminescent bacteria that were cultured to logarithmic growth phase and diluted 100 times with M9 medium of pH = 6.2, and then incubated at 37 °C for 6 h. The results showed that the RLU values of bacteria that adhered to the surface of aluminum, zinc and glass were 1197.2 ± 117.3, 802.0 ± 60.5 and 1010.5 ± 74.2, respectively. The adhesion to zinc was weaker than the other materials, and the adhesion to aluminum and glass was similar (*p* = 0.0184). Due to the characteristics of the materials, PE gloves, medical gauze and medical latex gloves had strong bacterial adhesion properties and the RLU values were 5438.2 ± 473.8, 4075.0 ± 255.0 and 6506.0 ± 323.7, respectively. The order of bacterial adhesion to the six materials as determined by the bioluminescence method is shown in Fig. 3A and was as follows: medical latex gloves > PE gloves > medical gauze > metal aluminum > glass > metal zinc. In addition, the traditional CFU counting method also showed that the three materials of aluminum, zinc and glass were in the same order of magnitude, and that the number of bacterial was about 10^6^ cells/mL. For the three materials with strong bacterial adhesion, PE gloves, gauze and latex gloves, the number was about 10^8^ to 10^9^ cells /mL (Fig. 3B, C). The results of the bioluminescence and CFU counting methods correlated with each other and the difference of bacterial adhesion between materials was obvious. For the materials with similar adhesions, bioluminescence detection was more rapid than CFU counting. In addition, bioluminescence detection was not easily affected by human error, the data were objective and the degree of quantification was high. Furthermore, the bioluminescence method also had the incomparable advantages of intuitionistic imaging observation and adhesion evaluation of multiple parts of the special-shaped materials.

### Effect of Triton X-100 on the growth and bioluminescence of the PAO1-*lux* strain

**Figure 4:**
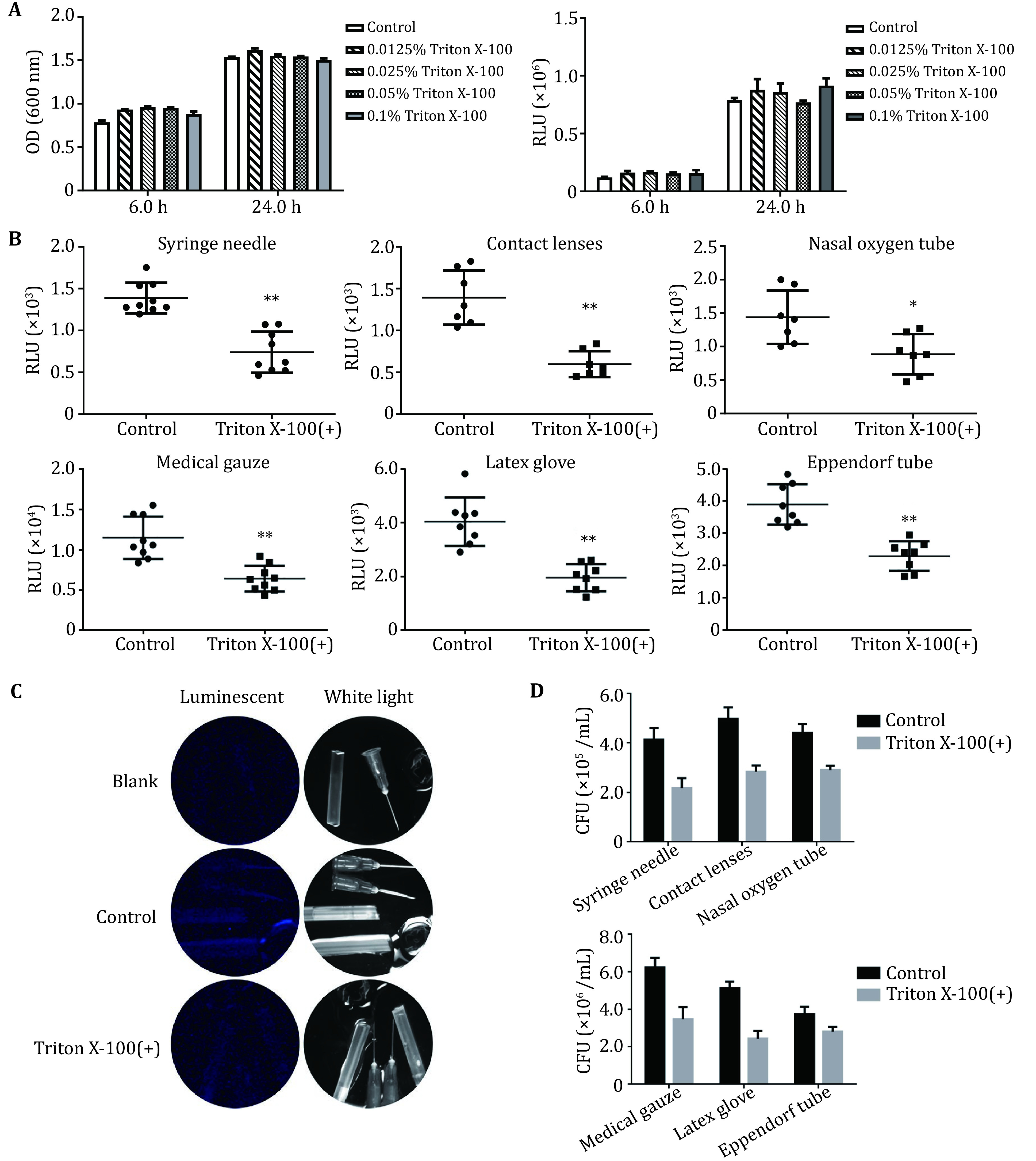
Determination of bacterial adhesion on the materials surface after Triton X-100 treated. **A** Effect of Triton X-100 in different concentrations on the growth and bioluminescence of the PAO1-*lux* strain. The PAO1-*lux* strain was cultured in LB liquid medium containing 0.1%, 0.05%, 0.025% or 0.0125% Triton X-100. The OD at 600 nm and RLU values were measured after 6 h and 24 h. **B** The changes of bacterial adhesion on six kinds of medical device materials’ surfaces were determined by bioluminescence method after Triton X-100 treatment. **C** Imaging of bacterial adhesion on the irregular materials’ surfaces by a Bio-Rad imaging system under white light and luminescent modes. **D** The changes of bacterial adhesion on six kinds of medical device materials' surfaces were determined by CFU counting method after Triton X-100 treatment

In order to detect the change of bacterial adhesion property of the material after different treatments (*e.g*., surfactant treatment or material modification) using bioluminescence detection, the sample to be tested was treated with surfactant Triton X-100 before the experiment to reduce the adhesion of bacteria to the material. LB liquid media containing 0.1%, 0.05%, 0.025% or 0.0125% Triton X-100 were used to dilute the bacteria in logarithmic phase by 100 times, which were then added to the tube for subculture (37 °C, 180 r/min). After 6 h and 24 h, the bacterial solutions were added to a 96-well plate, and the OD at 600 nm and RLU were measured and compared. As shown in Fig. 4A, Triton X-100 had no adverse effect on the growth and bioluminescence of the PAO1-*lux* strain in the concentration range of 0.0125% to 0.1%. Compared with the control group, the test groups had no significant differences in OD and RLU values. The results suggested that 0.1% Triton X-100 could be selected to pretreat material surfaces.

### Evaluation of bacterial adhesion on materials’ surfaces after Triton X-100 treatment

To further expand the application of the bioluminescent strain in the evaluation of bacterial adhesion on the surfaces of medical devices, screening of low adhesion or anti-adhesion medical materials and other fields, the surfactant Triton X-100, which has been shown to inhibit bacterial adhesion and does not affect the growth of the PAO1-*lux* strain, was selected to verify the reliability of bioluminescence detection. Six kinds of medical devices, including syringe needles, contact lens, medical gauzes, nasal oxygen tubes, latex gloves and PVC plastic tubes (Eppendorf tubes), were pretreated with PBS containing 0.1% Triton X-100. The bacterial solution cultured to logarithmic phase was diluted 100 times with M9 medium (pH 6.2), then coated on the surface of the pretreated (test group) or untreated (control group) material, and incubated at 37 °C for 6 h. The adherent bacteria were recovered by ultrasonic cleaning and determined by bioluminescence and CFU counting methods. The results in Fig. 4B show that the RLU values of the six samples in the test group measured by bioluminescence decreased significantly compared with the materials without surfactant treatment, with decline rates of 46.7% for syringe needles, 38.4% for contact lenses, 47.4% for medical gauze, 38.4% for nasal oxygen tubes, 53.0% for latex gloves, and 36.9% for EP tubes. As shown in Fig. 4D, the CFU counting method also obtained similar test results. After surfactant treatment, the decrease rates of bacterial adhesion of six materials were 47.6%, 43.0%, 44.4%, 34.1%, 52.6% and 25.0%, respectively. In addition, for irregular-shaped devices or materials, such as syringe needles, catheters and contact lenses, the bacteria that adhered to their surfaces could be visually photographed by the Bio-Rad imaging system. As shown in Fig. 4C, compared with the blank control group without bacterial incubation, the materials without surfactant treatment showed significant accumulations of bioluminescent signals after incubation with bacterial solution and the bioluminescence of materials pre-treated with Triton X-100 decreased significantly. Due to the different sensitivity of the detection equipment, it was more objective and accurate using the microplate reader to collect the RLU values to quantitatively determine the bacterial adhesion.

## DISCUSSION

*P. aeruginosa*, a representative strain of medical device pollution and nosocomial infection, has universal applicability in the evaluation of bacterial adhesion. The adhesion mechanism of bacteria on abiotic surfaces is usually related to the early formation of biofilm, so the evaluation of bacterial adhesion and the prevention and treatment of anti-adhesion are also generally applicable to different kinds of bacteria. In the screening experiment of anti-bacterial adhesion polymers, *P. aeruginosa*, *Staphylococcus aureus* and *Escherichia coli* all showed similar results (Hook *et al*. 2012), but there was also an abnormal situation that some bacteria did not change their adhesion to surfactants (Ishikawa *et al*. 2012). Bioluminescent microorganisms or *lux* reporter system can be applicable as biosensors for detection of water quality, lethality testing, for the discovery of anti-toxin deposits and pathogens in sustenance (Nyhan *et al*. 2020; Park *et al*. 2018). Since lux luciferase reporter proteins require molecular oxygen for catalysis, most of them are used in aerobic environment (Bruckbauer *et al*. 2015; Pribaz *et al*. 2012; Wang *et al*. 2018b), but there are few reports on their application in anaerobic microorganisms, such as *Clostridium perfringens* (Phillips-Jones 1993), *Mycobacterium tuberculosis* (Cho *et al*. 2007), and in anaerobically cultured *Escherichia coli* model organisms (Golding *et al*. 2008; Guglielmetti *et al*. 2019). The recombinant bioluminescent PAO1-*lux* strain used in this study contained the luciferase gene *lux*, which was integrated into the bacterial chromosome in the form of a single copy. Compared with the strain expressing *lux* through a plasmid, the PAO1*-lux* strain with *lux* integrated into the bacterial chromosome avoided the copy number effect and had more stable bioluminescent properties (Shah and Naseby 2014). Moreover, the complete *lux* gene could be expressed with the growth of bacteria and without adding exogenous substrate (Flemming *et al*. 1994). In addition, the results of this study showed that insertion of the *lux* gene had no adverse effects on the growth, phenotype or adhesion of PAO1 even though it has previously been reported that the insertion of the *lux* gene might affect the surface properties and transport ability of some bacteria (Chen *et al*. 2008). Furthermore, the properties of the bioluminescent strain in the process of planktonic or sessile growth were consistent with those of the standard PAO1 strain.

To establish a set of standard bioluminescence methods for detecting the number of adherent bacteria, it is necessary to investigate their accuracy, sensitivity and equivalence with traditional methods. In the correlation analysis of CFU counting with bioluminescent signal, the limit of detection for the bioluminescent assay was found by using multiple dilutions of the bacterial solution in logarithmic phase and it was found that the RLU decreased as the number of living bacteria decreased. The lowest threshold for this method was about 10^3^ bacteria per mL and the sensitivity of this method was consistent with that reported in the literature (Shah and Naseby 2015), which meet the needs of general detection. In a previous study of using recombinant *E. coli* with a green fluorescent protein (GFP) gene marker to detect the adhesion of catheter bacteria, the minimum number of bacteria detected was 1.2 × 10^2^ CFU (AlLuhaybi *et al*. 2015), which indicates that in terms of detection sensitivity, GFP fluorescence signal has certain advantages over the *lux* gene bioluminescent signal. However, fluorescence detection requires more advanced instruments and the persistence and stability of the fluorescence signal is weak (Wang *et al*. 2018a).

In terms of detection range, there was a significant linear correlation between the number of living bacteria and the bioluminescence (*R*^2^ = 0.969) in a previous study using *P. aeruginosa* to evaluate the antibacterial effect of wound dressings, when the bioluminescent MCS5-lite strain grew in planktonic state and reached the exponential phase, the CFU was in the range of 10^7^ to 10^9^ (Thorn *et al*. 2007). The method used in our study also maintained a good linear relationship at a lower range (10^4^ to 10^7^) of living bacteria and the detection range was wider. Therefore, the detection range was suitable for the detection of bacteria on various materials, such as latex gloves and medical gauzes that have high adhesion as well as glass and metal zinc, which have low adhesion. The characteristics of four different methods for bacterial adhesion testing are shown in Table 1.

**Table 1:** Comparison of bacterial adhesion testing methods

	CFU counting	Crystal violet staining	ATP	Bioluminescence
Accuracy	RSD ≤ 30%	RSD ≤ 30%	RSD ≤ 30%	RSD ≤ 15%
Range	1 to 10^8^ cells	10^4^ to 10^9^ cells	10^2^ to 10^8^ cells	10^3^ to 10^8^ cells
Sensitivity	1 to 10^2^ cells	10^3^ to 10^4^ cells	10^2^ cells	10^3^ cells
Specificity	Strong	Weak (Dye can be adsorbed to the abiotic materials)	Weak (Non-microbial ATP can interfere with detection)	Strong
Testing time	2 d	3–4 h	1–2 h	1–2 h
Testing steps	4 steps	4 steps	3 steps	2 steps
Invasive detection	No	Yes	Yes	No
Real time detection	No	No	No	Yes
Applicability for materials of irregular shape	No	No	Yes	Yes
Applicability for batch testing	No	Yes	Yes	Yes
Testing equipment requirements	Low	Medium	High	High
Reference	Meireles *et al*. 2015; Pantanella *et al*. 2011	Honraet *et al*. 2005; Martin and An 2000; Stepanovic *et al*. 2000	Dostalek and Branyik 2018; Harber *et al*. 1983; Robrish *et al*. 1978	

Most studies on the determination of bacterial adhesion on the surface of materials *in vitro* using bioluminescent strains usually only researched a single material and the emphasis was on surface modifications, such as nano-material modification of medical sutures (Serrano *et al*. 2015) and coating treatment of catheter materials (Yu *et al*. 2017). In our study, the bioluminescence method was comprehensively used in the detection of bacterial adhesion on various materials for the first time. The materials involved in the test included polystyrene microplates, polypropylene tubes, polyethylene plastic gloves, glass and metal aluminum, which are commonly used in the laboratory, as well as medical equipment products, such as syringe needles, contact lenses, medical gauzes, nasal oxygen tubes, latex gloves and metal zinc. Through the measurement of bioluminescent signal, bacterial adhesion to different materials and different treatments of the same material could both be compared and evaluated. The recombinant strain has excellent stability of the bioluminescence, which can be applied to a variety of research fields, and can be used to supplement or replace the traditional CV staining and CFU counting method. The equivalence between the new method and the traditional method showed that the bioluminescence not only achieved the same detection results as the traditional method, but also had irreplaceable advantages over the traditional method. The bioluminescence method took only two days from the bacterial culture to the end of the detection and the manual operation time was less than two hours. In addition, multiple samples could be assessed in parallel at the same time. Moreover, the bioluminescence method also has the advantages of real-time monitoring of samples, intuitive detection of the number of bacteria adhering to different parts of special-shaped materials, a non-invasive testing process and sustainable culture of adherent bacteria after testing. Because of the obvious advantages of this method, it has been widely used in the research of medical devices implanted *in vivo*. For example, the bioluminescent *P. aeruginosa* PAO1 Tn7: Plac-*lux* was used to detect infections on catheters with an anti-adhesion and antimicrobial peptide coating. In addition, bioluminescence detection can directly show the infection process of bacteria in mice, but the traditional CFU counting method can only be used to detect the anti-bacterial adhesion performance of catheters *in vitro*; however, the bioluminescent characteristics have not been deeply studied (Yu *et al*. 2017). Combined with the experimental data *in vitro* of our study, the recombinant PAO1-*lux* strain has the application value for the comprehensive evaluation of anti-adhesion properties of medical materials both *in vitro* and *in vivo*.

**Figure 5:**
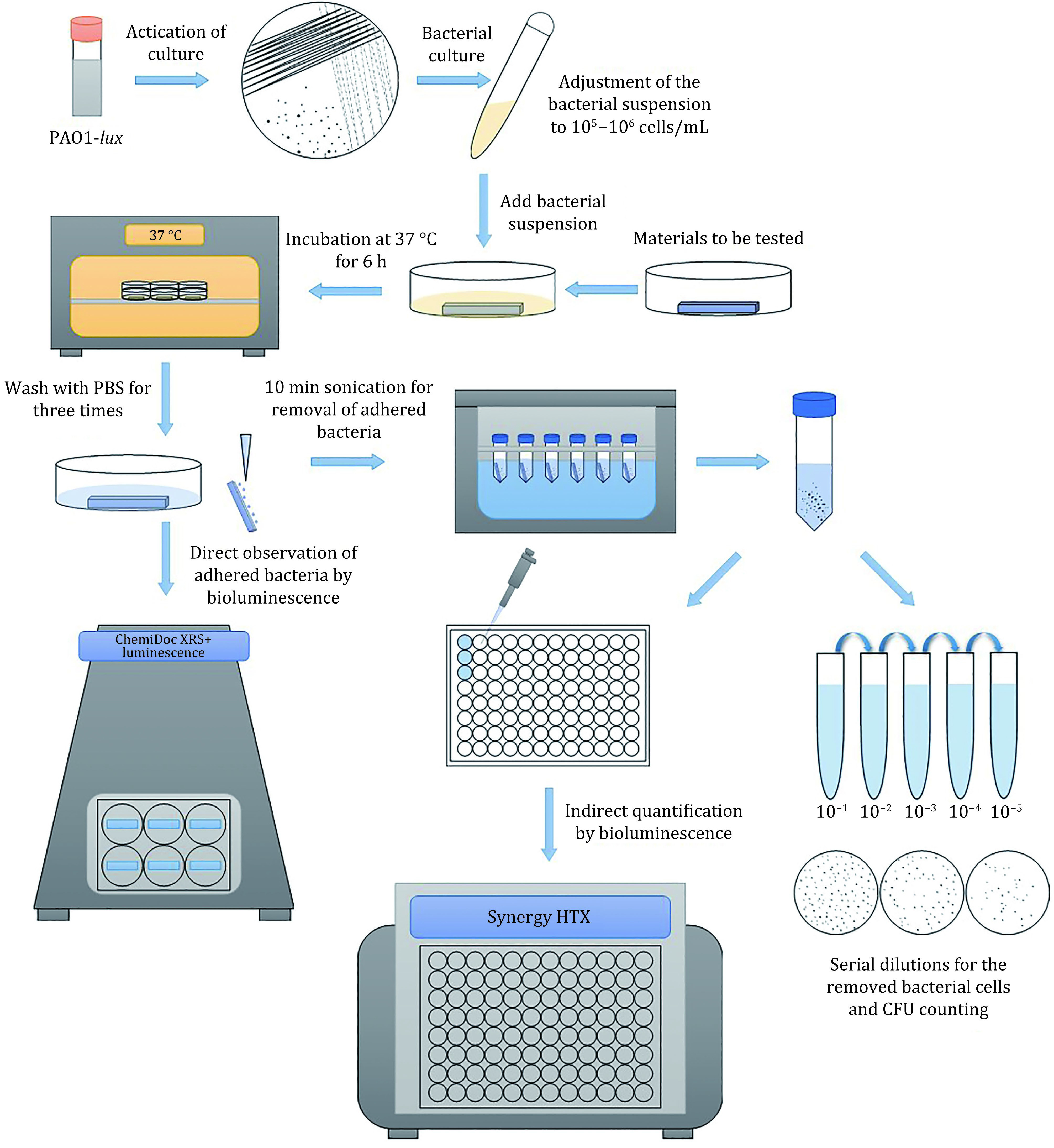
Schematic diagram of the procedure for assaying bacterial adhesion using the PAO1-*lux* strain

In order to establish an easy-to-operate detection procedure, this study, combined with the traditional methods for the recovery of adherent bacteria, made use of the correlation between bioluminescence and the CFU counting method to rapidly quantify the number of adherent bacteria. For the detection of bacterial adhesion on the material surface, the following standard procedures were preliminarily formed: (1) The surface area of tested samples was unified before detection; (2) The test concentration of bacteria could be appropriate to control at 10^5^ to 10^6^ CFU per mL; (3) The adhesion assay was carried out in M9 medium at pH 6.2 with low nutrient content and the incubation time was 6 h; (4) The cleaning procedure was washing three times with PBS and collecting the adherent bacteria by ultrasonic cleaning for 10 min; (5) The RLU value of the same batch of bacterial culture was measured and the linear correlation curve between the RLU and CFU values was established. The schematic diagram of the procedure for assaying bacterial adhesion using the PAO1-*lux* strain is shown in Fig. 5. In addition, this method can also be used to evaluate the changes of bacterial adhesion on irregular or special-shaped materials before and after surface treatment. Furthermore, this method can provide reference data and suggestions for the screening of antibacterial and anti-adhesive materials.

## CONCLUSION

In the present study, a recombinant bioluminescent *P. aeruginosa*, PAO1-*lux* strain, was constructed to evaluate the initial bacterial adhesion on the surfaces of medical devices or materials *in vitro*. Quantitative determination of bacterial adhesion to the surface of a variety of materials and medical devices was carried out by traditional CFU counting and bioluminescence assays. The results revealed that no significant difference in the evaluation of bacterial adhesion was observed by the two methods, which demonstrated the efficiency of using the bioluminescence assay as a rapid method for the quantitative determination of bacterial adhesion. Compared with traditional CFU counting, the bioluminescence assay had obvious advantages in detection time, operation steps, batch testing, real-time monitoring and detection on materials with complex structures. Accordingly, the bioluminescent PAO1-*lux* strain was found to be a versatile tool for applying a simple, rapid and sensitive method in bacterial adhesion studies as well as other related fields. As a supplement and replacement for traditional microbiological testing procedures, this method has the potential to be used for further studies on bacterial adhesion to the surface of inanimate objects or living tissues. With the bioluminescent assay’s wide applicability, it is expected to become a standard method for the detection of bacterial adhesion and the screening of anti-adhesion materials.

## MATERIALS AND METHODS

### Materials and reagents

The experimental materials used for the bacterial adhesion test included 96-well polystyrene microplates (Costar, USA), cover glass slides (2 cm × 2 cm), polypropylene Eppendorf tubes, polyethylene plastic gloves, latex gloves, aluminum slices and metal zinc, which were all provided by the laboratory. The medical device materials included syringe needles (Longkangxin Medical Equipment Co., Ltd. Shaanxi), corneal contact lens (Hydron, Shanghai), medical absorbent gauzes (Hongda medical materials Co., Ltd. Xinxiang), disposable nasal oxygen tubes (Nolida medical Equipment Co., Ltd. Shijiazhuang) and rubber surgical gloves (Huaxiang medical equipment factory, Yangzhou). The bacterial culture media were LB medium (10 g tryptone, 5 g yeast extract and 10 g NaCl per liter) and M9 minimal medium, which was composed of 12.8 g Na_2_HPO_4_**·**7H_2_O, 3 g KH_2_PO_4_, 0.5 g NaCl, 1 g NH_4_Cl and 4 g glucose (0.4%) per litre as well as 2 mmol/L MgSO_4_ and 100 μmol/L CaCl_2_.

### Bacterial strains and bacterial cultures

The bacterial strains and plasmids used in this study are listed in Table 2. Two bacterial reference strains (PAO1 and PAO1-*lux*) were used to investigate the adhesion properties of the materials *in vitro*. The PAO1 strain was kindly provided by Professor Kangmin Duan (Department of Medical Microbiology, University of Manitoba, Canada). The bioluminescent *P. aeruginosa* strain (PAO1-*lux*) was constructed by integrating the *luxCDABE* luciferase gene into the bacterial chromosome. The PAO1 and PAO1-*lux* strains were routinely grown in Luria Bertani (LB) medium broth or on LB agar unless otherwise stated. Before the experiment, a single colony was selected and cultured in LB liquid medium at 37 °C for 16 to 18 h on an orbital shaker at 180 r/min. Then 100 μL of the bacterial solution was added to 10 mL of fresh medium and the logarithmic growth phase was tested by detecting the optical density (OD) at 600 nm every 2 h.

**Table 2:** Reference strains and plasmids used in this study

Bacterial strain or plasmid	Genotype or phenotype	Source or reference
*E. coli* strains		
DH5α	F^-^ *mcrA Δ*(*mrr-hsdRMS-mcrBC*)φ80 *lacZ ΔM15 ΔlacX74 deoR recA1 endA1 araD139 Δ*(*ara leu*)*7697 galU galK λ*^-^ *rpsL nupG*	Invitrogen
*P. aeruginosa* strains		
PAO1	Wild type, standard strain	This study
PAO1-*lux*	Recombinant strain	This study
Plasmids		
pMS402	Expression reporter plasmid carrying the promoterless *luxCDABE*; Kn^r^, Tmp^r^	Liang *et al*. 2008
pKD-*algU*	pMS402 containing *algU* promoter region, Kn^r^, Tmp^r^	This study
Mini-CTX	Integration plasmid, Tet^R^	Hoang *et al*. 2000
pRK2013	Broad-host-range helper vector; Tra^+^, Kn^r^	Schweizer 1992

### Construction of bioluminescent PAO1-*lux* strain

The bioluminescent PAO1-*lux* strain was constructed by integrating the *luxCDABE* luciferase gene into the bacterial chromosome. Briefly, the *algU* gene promoter sequence of *P. aeruginosa* containing the restriction site XhoI was obtained by PCR and named PalgU. The PalgU sequence was digested and ligated to the pMS402 plasmid, which was a luciferase reporter gene vector containing *luxCDABE* without a promoter. The reporter gene vector containing the PalgU promoter was named pKD-algU. The Mini-CTX vector plasmid was linked with pKD-algU at the same time and the vector containing the genomic integration site was obtained, which was named CTX-pKD. The *E. coli* with the recombinant plasmid CTX-pKD, the receptor bacteria of the PAO1 standard strain and the *E. coli* containing the assistant plasmid pRK2013 were each separately cultured on LB agar. The recombinant bioluminescent strain PAO1-*lux* was obtained by a three-parent strain hybridization experiment.

### Monitoring of the growth and bioluminescence of the PAO1 and PAO1-*lux* strains

The growth and bioluminescence of the PAO1 and PAO1-*lux* strains were monitored under planktonic or sessile conditions in LB or M9 media. For monitoring under planktonic conditions, the strains were sub-cultured to an OD at 600 nm of 0.5 and grown in flasks at 37 °C with shaking at 180 r/min. To monitor bioluminescence under sessile conditions, the strains in logarithmic phase cultures were diluted 100 times and added to 96-well white and transparent microplates (200 μL/well). The microplates were then incubated at 37 °C. The OD and bioluminescence were simultaneously measured every 0.5 h using a multi-functional microplates reader (Synergy HTX, Bio-Tek). Data are reported as relative light units (RLU) and wells with sterile saline solution were used to determine the background value, which was subtracted from the bioluminescence values from the wells with bacteria.

### Bioluminescence stability of the PAO1-*lux* strain

The recombinant bioluminescent strain and the standard strain were inoculated on LB agar. The bioluminescent stability of PAO1-*lux* strain over time was monitored using an imaging system (ChemiDoc XRS+, Bio-Rad) under white light and luminescent modes. The PAO1-*lux* and PAO1 strains were cultured to logarithmic phase and two-fold serial dilutions of the bacterial suspension in saline solution were prepared from approximately 10^4^ to 10^7^ CFU per mL. The serial dilutions were inoculated into a 96-well plate and the 96-well plate was photographed with Bio-Rad imaging system in white light mode and luminescent mode. The 96-well plate was observed at the 1st, 3rd, and 7th day by a multi-mode microplate reader (Synergy HTX, Biotek). The OD at 600 nm and luminous intensity (RLU) were detected simultaneously and established the linear correlation curve between them. The PAO1-*lux* strain was cultured for ten consecutive generations and each generation was preserved. The ten generations of the PAO1-*lux* strain were cultured to stationary phase and serial dilutions were inoculated into a 96-well plate for imaging and detecting.

### Bacterial adhesion assays in 96-well microplates

The bacterial adhesion assays were performed in 96-well polystyrene microplates as previously described with a few modifications. Overnight cultures of the PAO1-*lux* strain were diluted 1:100 in LB or M9 medium and incubated until the OD at 600 nm reached 0.5. These cultures were continuously cultured at 37 °C for 24 h under different nutritional conditions (LB or M9 medium) and different pH conditions (pH 6.2 or 7.2). The OD values and RLU values of the 96-well microplate were detected by a microplate reader every 0.5 h and the bioluminescence pictures at a single time point were collected using a Bio-Rad imaging system.

### Crystal violet staining method

Bacterial adhesion was analyzed using the crystal violet (CV) assay following the protocol with some modifications (Wilson *et al*. 2017). The bacterial suspension was inoculated into 96-well microplates and incubated for 6, 12, 24 and 36 h at 37 °C. The non-adherent cells and media were removed. The bacteria were washed three times to remove any remaining non-adherent bacteria using sterile phosphate-buffered saline (PBS, pH 7.2). A 10% methanol solution was then used to fix slime and adherent organisms for 20 min. The wells were then washed using 50% ethanol three or four times. The staining of adherent bacteria was then performed using 200 μL of 0.1% (*w*/*v*) crystal violet in distilled water for 20 min. The excess stain in microplate wells was removed by rinsing the plates with running distilled water followed by air-drying for 15 min at room temperature. To release the dye adsorbed by the adherent bacteria, a solubilization step was performed by adding 200 μL of 95% ethanol per well followed with an agitation of 5 min at 120 r/min. Then the OD of each well was measured at 570 nm using a microplate reader (Synergy HTX, Biotek). The data are reported as the absorbance at 570 nm of bacterial samples with the absorbance of the blank well that did not have sterile media being subtracted. These values were divided by the absorbance of the sterile media, which were used as controls. Triplicate measurements were performed and were repeated three times.

### Bacterial adhesion assay on the surface of different materials

The method reported in the reference carried on the bacterial adhesion treatment to the surface of the test sample or medical device product (Yu *et al*. 2017). For the different materials, including metal aluminum, metal zinc, glass, polyethylene plastic gloves, medical gauzes and medical latex gloves, the samples to be tested were cut into thin slices with the size of 2 cm × 2 cm (*N* = 3) before the experiment. The samples that could not be autoclaved were soaked in 75% ethanol for 20 min and cleaned with PBS thrice after removing the ethanol. For the medical device products, including syringe needles, corneal contact lenses, medical gauzes, nasal oxygen tubes and rubber surgical gloves, there were three groups: the surfactant pretreatment group, the control group without surfactant treatment and the blank control group without bacteria. The prepared bacterial culture of the PAO1-*lux* strain (diluted 1:100 from the logarithmic phase) was added to the 6-well plate or petri dish containing the samples to be tested. It was ensured that all samples were completely immersed under the liquid surface. They were then cultured at 37 °C for 6 h. The samples to be tested were taken out, rinsed with sterile PBS three times to wash off the planktonic bacteria. Then each sample was put into an Eppendorf tube containing 2 mL of sterile PBS and cleaned 10 min by ultrasonic cleaning. 200 μL of the recovered bacterial solution was added to a 96-well microplate to determine the RLU. At the same time, the recovered bacterial solution of each sample was serially diluted and 10 μL of the diluent was dripped on a culture plate. The CFU were counted after overnight culture.

### Statistical analysis

GraphPad Prism 6 (GraphPad Software, San Diego, CA, USA) was used for statistical analyses of the results regarding the bacterial adhesion assay and the RLU values under planktonic and sessile conditions. Student *t-*tests and analysis of variance were used to determine the statistical significance between means. Treatment effects were separated using Bonferroni’s and Tukey’s multiple comparison post-tests. Statistical significance was accepted from a *p* value of <0.05.

## Conflict of interest

Lu Wang, Xinhua Qiao, Lei Gao, Chang Chen and Yi Wan declare that they have no conflict of interest.
